# Decreased Levels of Circulating Cancer-Associated Protein Biomarkers Following Bariatric Surgery

**DOI:** 10.1007/s11695-016-2321-y

**Published:** 2016-08-15

**Authors:** John Edward Farey, Oliver M. Fisher, Angelique J. Levert-Mignon, Patrice M. Forner, Reginald V. Lord

**Affiliations:** 1 Department of Surgery, University of Notre Dame Australia, School of Medicine, Sydney, Darlinghurst, NSW Australia; 2 Gastro-Oesophageal Cancer Research Program, St Vincent’s Centre for Applied Medical Research, Darlinghurst, NSW Australia

**Keywords:** Bariatric surgery, Neoplasms, Biological markers, Gastrectomy, Neoplasms/prevention and control, Laparoscopy

## Abstract

**Background:**

Epidemiological studies have identified obesity as a major risk factor for cancer in humans, and trials have demonstrated a significant reduction in the incidence of cancer after bariatric surgery. The rapidity of weight loss after bariatric surgery provides an opportunity to identify the molecular changes associated with effective obesity treatment. Indirectly, this may provide some insights into the mechanisms that drive the association between obesity and cancer. We sought to measure circulating cancer-associated proteins before and after laparoscopic sleeve gastrectomy (LSG).

**Methods:**

We prospectively enrolled 15 patients undergoing LSG. Thirty-four plasma protein biomarkers thought to be associated with cancer processes were analyzed at baseline and following successful weight loss at 12 weeks using a multiplex bead-based assay.

**Results:**

Mean excess body weight loss was 44 % at 12-week follow-up. After LSG, a significant reduction in circulating plasma levels was observed for half (17/34) of the proteins assessed: VEGF-A, VEGF-C, VEGF-D, endoglin, PLGF, sFASL, IGFBP-1, IL-18, prolactin, EGF, TGFα, sCD40L, IL-18, TNFα, IL-6, HB-EGF, and PAI-1. Nonsignificant decreases were found for the remaining proteins.

**Conclusions:**

Circulating cancer-related biomarker levels were reduced by surgical weight loss, and this benefit was achieved as early as 3 months after operation. The observed reduction in cancer biomarkers may be related to the reported decrease in cancer incidence following bariatric surgery.

## Introduction

Obesity is one of the key issues facing human health, threatening to reverse the incremental gains in life expectancy accrued in the twentieth century [1]. This year, the World Health Organization estimates that two billion adults worldwide are now either overweight (BMI > 25 kg/m^2^) or obese (BMI > 30 kg/m^2^). Approximately 39 % of the world’s population thus has a moderate to severe increase in their risk of acquiring obesity-related comorbidities, of which type 2 diabetes mellitus (T2DM) is the paradigm illness [2]. Large epidemiological studies have demonstrated a gradient between excess body weight and the relative risk of cancer [3], suggesting a causal association between increased adiposity and the development of cancer. Obesity is now considered a major etiological factor in cancers of the esophagus (adenocarcinoma), breast (postmenopausal), endometrium, colon and rectum, kidney, pancreas, thyroid, and gallbladder [4]. It is estimated that by 2030, obesity will be the major contributor to the 50 % increase in the worldwide number of cancer cases, overtaking tobacco use [5].

Bariatric surgery provides effective and enduring therapeutic weight loss [6, 7]. Population-based studies have shown a reduction in cancer incidence and mortality following bariatric surgery [8–10], suggesting a causal link between excess adiposity and cancer. Proposed mechanisms leading to a tissue microenvironment favorable for the development of cancer in the obese include a chronic, low-grade inflammatory state, increased expression of proangiogenic factors, secondary cell proliferation by excess sex hormones and obesity hormones (adipokines), hyperinsulinaemia, and increased oxidative stress [11, 12].

Previous bariatric surgery studies using serial blood sample analysis have reported on multiple factors associated with cardiovascular risk, insulin resistance, metabolic profile, and inflammation [13–16]. Despite the interest in bariatric surgery and reduced cancer outcomes, relatively few reports in humans have evaluated the serological changes in cancer-related factors after bariatric surgery [17–21].

This study was undertaken to examine whether the blood levels of cancer-related biomarkers are altered by surgical weight loss. As well as contributing to the scientific basis of bariatric surgery, it was anticipated that the results might provide some insights into the molecular pathways linking obesity with cancer.

## Materials and Methods

### Participants

We recruited obese participants from a single center presenting for bariatric surgery to a single surgeon (RVL) in a tertiary referral hospital. Inclusion criteria were age > 18 years and BMI > 30 kg/m^2^ with obesity comorbid illness, or BMI > 40 kg/m^2^. Exclusion criteria were any past or present history of benign or malignant tumor or an inflammatory or autoimmune disorder, and regular use of anti-inflammatory or corticosteroid medications. Two study visits were scheduled: the first at baseline prior to bariatric surgery and the second at the routine 12-week postoperative follow-up.

A detailed clinical assessment, including comorbidities and medication use, was made each visit. Anthropometric data including height, weight, BMI, and excess body weight were measured with patients barefoot. BMI was calculated as weight/height × height (kg/m^2^). Excess body weight (EBW) was calculated as weight above the ideal BMI of 25 kg/m^2^ and converted to kilos. Clinical data including comorbidities and medication use was extracted from patients’ medical records.

IRB ethical approval was provided by the St Vincent’s Hospital Human Research Ethics Committee (reference number HREC/14/SVH/291), and informed consent was obtained from all individual participants included in the study. All procedures performed in studies involving human participants were in accordance with the ethical standards of the institutional and/or national research committee and with the 1964 Helsinki declaration and its later amendments or comparable ethical standards.

### Bariatric Operation

A conventional laparoscopic sleeve gastrectomy was performed in all patients. In brief, the patient was placed in the lithotomy position and pneumoperitoneum created using five port sites. The liver was elevated using a Nathanson liver retractor, and the vessels along the greater curvature were divided using the blunt tip LigaSure™ device (Covidien, Medtronic, Dublin, Ireland), from a distance of 4–6 cm lateral to the pylorus to a point 1–2 cm lateral to the gastroesophageal junction. A hiatus hernia, if present, was repaired with closure of the diaphragmatic opening using interrupted 0 Ethibond (Ethicon Inc., Somerville, USA) posterior crural sutures. The stomach was divided using a motorized linear cutting stapler device (Echelon Endopath™, Ethicon, Somerville, NJ) over a 32-French bougie for females or a 36-French bougie for males. The resected stomach was removed through the large (15 mm) port site. A 10-French Redivac™ drain was left adjacent to the staple line, and the wounds were closed with 0-PDS and 4/0 undyed monocryl. There was no operative mortality and no morbidity.

### Blood Collection and Preparation

Fasting blood samples were collected at each study visit. The blood was spun in a benchtop centrifuge at 1400×*g* for 10 min. The plasma was then transferred into cryotubes and stored at −80 °C until analysis.

### Cancer-Associated Biomarker Measurement

Two commercially available multiplex bead-based kits (BioRad, Hercules, CA, USA) were used to measure concentrations of 34 biomarkers associated with the development of cancer in humans. The first assay, the Bio-Plex Pro Human Cancer Biomarker Panel 1 (171-AC500M), was used to measure 16 receptors, cytokines, chemokines, growth factors, and hormones, including soluble epidermal growth factor receptor (sEGFR), FGF-basic, follistatin, G-CSF, HGF, soluble human epidermal growth factor 2 (sHER-2/neu), soluble interleukin-6 receptor alpha (sIL-6Rα), leptin, osteopontin, PECAM-1, PDGF-AB/BB, prolactin, SCF, sTIE-2, soluble vascular endothelial growth factor receptor (sVEGFR)-1, and sVEGFR-2. The second assay, the Bio-Plex Pro Human Cancer Biomarker Panel 2 (171-AC600M), was used to measure 18 cytokines, chemokines, growth factors, and ligands for receptors in Panel 1, including angiopoietin, soluble CD40 ligand (sCD40L), epidermal growth factor (EGF), endoglin, sFASL, heparin-binding EGF-like growth factor (HB-EGF), insulin-like growth factor binding protein-1 (IGFBP-1), interleukin (IL)-6, IL-8, IL-18, plasminogen activator inhibitor-1 (PAI-1), placental growth factor (PLGF), transforming growth factor alpha (TGFα), TNF-α, urokinase plasminogen activator (uPA), vascular endothelial growth factor (VEGF)-A, VEGF-C, and VEGF-D.

Plasma protein biomarker levels were quantified at the Australian Proteome Analysis Facility at Macquarie University, Sydney, Australia, according to a an optimized process described previously [22], run in duplicate on 96-well plates using 50 μL of neat plasma per assay plate. The standards and samples were dispensed using a robotic liquid handling workstation (epMotion 5075, Eppendorf, Germany); plates were washed with Bio-Plex Pro II wash station (Bio-Rad, CA, USA). Samples were analyzed and standard curves [log(*x*) − linear(*y*)] were generated using the Bio-Plex Manager v5.0 software (Bio-Rad, CA, USA).

### Statistical Analysis

Where necessary, data were log2-transformed to fit normal distribution. Differences between continuous variables measured at baseline and at 12-week follow-up were analyzed using paired *t* tests. Categorical variables were compared using McNemar’s test. Correlation analysis of plasma levels of measured biomarkers and patient BMIs was performed through linear regression as well as analysis of Pearson’s product moment correlation coefficient. A value of *p* < 0.05 was considered statistically significant. Data are presented as mean with standard deviation (SD) unless denoted otherwise. All analyses were performed using R version 3.2.2 (R Foundation for Statistical Computing, Vienna, Austria).

## Results

### Participant Characteristics

Fifteen patients (eight males, seven females) were recruited to this study. Patient demographics at baseline and at 12 weeks after laparoscopic sleeve gastrectomy are summarized in Table 1. The median values (not shown in Table 1) were similar to the mean values: mean age was 50.9 years (SD 11.9), with an average preoperative weight of 122.3 kg (SD 30.7 kg) and a resulting mean cohort BMI of 42.3 kg/m^2^ (SD 8.2 kg/m^2^). All but two patients, both of whom were morbidly obese (BMI > 40 kg/m^2^), had at least one obesity-related comorbid illness.

**Table 1:** Demographic, anthropometric, and obesity-related comorbid illness variables in obese participants at baseline and 12 weeks after laparoscopic sleeve gastrectomy

Variable	Obese group baseline	Obese group 12 weeks after surgery	*p* value
Demographics			
Male	8	8	–
Female	7	7	–
Age (years)	50.9 ± 11.9	50.9 ± 11.8	–
Anthropometric^a^			
Weight (kg)	122.3 ± 30.7	102.1 ± 24.4	< 0.001
BMI (kg/m^2^)	42.3 ± 8.2	35.3 ± 6.4	< 0.001
EBW (kg)	50.3 ± 26.4	30.03 ± 20.4	< 0.001
Weight loss (kg)	–	20.25	–
EBWL (%)	–	44 ± 11	–
Obesity-related comorbid illness^b^			
T2DM	6 (40 %)	2 (13 %)	0.11
Hypertension	7 (47 %)	4 (33 %)	0.35
Hyperlipidemia	5 (33 %)	4 (33 %)	0.13
OSA	12 (80 %)	10 (66 %)	0.09
GERD	4 (33 %)	1 (7 %)	0.02

Unless otherwise stated, data are mean ± SD

*OSA* obstructive sleep apnea, *T2DM* type 2 diabetes mellitus, *GERD* gastroesophageal reflux disease

^a^Comparisons are by paired *t* tests

^b^Comparisons are by McNemar’s test

### Weight Loss Following LSG at 12-weeks Follow-up

The median length of follow-up was 12 weeks (range 11–13 weeks). At last follow-up, mean weight loss was 20.25 kg (range 9.8–31 kg), with a mean reduction in BMI of 7.0 kg/m^2^ (SD 2.4) and a mean percent excess body weight loss (%EBWL) of 44 % (SD 11 %) (Table 1). There were reductions in all of the obesity-related comorbidities at 12-week follow-up, but only one of these was of statistical significance.

### Changes in Cancer-Associated Plasma Biomarkers Following LSG

Significant reductions were observed for 17 of the 34 biomarkers measured. These are summarized in Table 2, grouped according to their primary associations in the literature with the ‘Hallmarks of Cancer’ [14]. Significant reductions were observed for all members of the VEGF family (VEGF-A, VEGF-C, VEGF-D, and PLGF), but not their corresponding receptors (sVEGFR1, sVEGFR2). A significant reduction in all of the adipokines and proinflammatory biomarkers (IL-6, IL-18, TNFα, PAI-1, IL-8, and sCD40L), except leptin, was observed. Similar to the angiogenic biomarkers, the cytokine IL-6 was significantly reduced at follow-up, but not its corresponding receptor sIL-6Rα. All members of the epidermal growth factor family (EGF, TGFα, and HB-EGF) were significantly reduced, but neither of their related receptors (sEGFR and sHER2/neu) showed significant changes at follow-up. Expression levels of the 17 significantly reduced proteins at baseline and follow-up are shown in Fig. 1.

**Table 2:** Alterations in plasma levels of 34 cytokines, ligands, growth factors, hormones, and their receptors at baseline and 12 weeks after laparoscopic sleeve gastrectomy

Hallmark of cancer	Cytokines, ligands, growth factors, and hormones				Corresponding receptors			
	Variable	Baseline	12 weeks after LSG	*p* value	Variable	Baseline	12 weeks after LSG	*p* value
Angiogenesis	Vascular endothelial growth factor (VEGF) superfamily							
	VEGF-A	129.6 ± 26.7	107.7 ± 32.4	0.01	sVEGFR1	79.2 ± 29.9	85.8 ± 43.0	0.53
	VEGF-C	494.43 ± 84.4	434.6 ± 96.1	0.02	sVEGFR2	780.1 ± 275.7	707.5 ± 138.3	0.62
	VEGF-D	228.3 ± 33.0	206.5 ± 42.3	0.04				
	PLGF	50.7 ± 10.4	43.6 ± 11.0	0.02				
	Other vascular growth factors							
	Angiopoietin-2	540.1 ± 242.2	531.7 ± 339.0	0.47	sTIE-2	3293.9 ± 2602.2	2977.9 ± 2339.0	0.49
					Endoglin	294.4 ± 70.8	254 ± 80.5	0.03
	PDGF-AB/BB	91.7 ± 35.1	76.6 ± 19.5	0.26				
	G-CSF	87.7 ± 37.0	90.9 ± 47.3	0.96				
	FGF-basic	62.2 ± 17.7	63.5 ± 17.7	0.88				
Inflammation	Adipokines							
	IL-6	45.1 ± 10.2	37.1 ± 11.0	0.008	sIL-6Rα	121.3 ± 111.2	104.2 ± 111.6	0.69
	IL-18	88.6 ± 33.0	54.5 ± 58.7	0.003				
	TNFα	40.0 ± 8.1	34.1 ± 10.3	0.02				
	Leptin	122.0 ± 45.3	105.6 ± 21.2	0.34				
	Other proinflammatory biomarkers							
	PAI-1	3135.2 ± 1390.1	2251.0 ± 1266.4	0.003	uPA	194.8 ± 60.6	184.7 ± 62.1	0.24
	IL-8	10.9 ± 2.9	8.7 ± 2.7	0.005				
	sCD40L	106.1 ± 25.7	87.7 ± 27.2	0.01				
Cell proliferation	Epidermal growth factor family							
	EGF	17.7 ± 6.4	8.3 ± 12.1	0.003	sEGFR	579.6 ± 245.6	578.8 ± 96.0	0.55
	TGFα	47.6 ± 11.1	32.0 ± 40.7	0.03	sHER2/neu	67.3 ± 51.1	53.9 ± 30.4	0.31
	HB-EGF	26.1 ± 5.5	21.9 ± 6.2	0.01				
	Hormones associated with obesity and insulin resistance							
	Prolactin	959.6 ± 485.1	449.3 ± 593.5	0.004				
					IGFBP-1	1268.6 ± 421.9	717.8 ± 1023.8	0.02
	Other growth factors							
	Follistatin	309.0 ± 148.7	278.5.0 ± 104.2	0.72				
	HGF	320.9 ± 171.6	281.8 ± 79.3	0.7				
	SCF	41.8 ± 10.5	41.0 ± 9.4	0.89				
Cell adhesion	PECAM-1	289.4 ± 149.0	249.5 ± 96.6	0.3				
Apoptosis	sFASL	224.0 ± 36.6	196.6 ± 39.0	0.03				
Metastasis	Osteopontin	1912.5 ± 1330.0	1858.9 ± 1686.9	0.61				

The biomarkers are grouped according to their best match for one of the “Hallmarks of Cancer”after LSG; many are associated with more than one “Hallmark.” Data are mean ± SD. Comparisons are by paired *t* tests

## Discussion

In this study, we found a marked decrease in the levels of circulating human cancer-associated proteins following laparoscopic sleeve gastrectomy. Half (17/34) of the cancer biomarkers studied were significantly reduced, and levels of the remaining proteins had also decreased. This effect was achieved early, at only 12 weeks after operation, which is presumably due to the rapidity of weight loss and amelioration of metabolic disease after effective bariatric surgery; by 12 weeks, the patients had already lost a mean 20 kg, equating to 44 % loss of excess body weight. We provide evidence demonstrating that bariatric surgery may significantly reduce expression of the molecular pathways thought to be associated with obesity and cancer. Whether this finding is of clinical significance is as yet unknown. Our results, however, provide insights as to how bariatric surgery may be an effective method of cancer prevention, as has been demonstrated in large clinical studies [8–10].

This study investigated a broad spectrum of biomarkers causally implicated in cancer development pathways, but of uncertain prognostic or diagnostic value. Our findings are in accordance with previously reported factors in the bariatric literature, including VEGF-A [17], IL-6 [21], TNFα [21], IL-8 [18], IL-18 [19], PAI-1 [20], sCD40L [23], and prolactin [24]. Some related protein families associated with specific molecular pathways are also novel findings here, including the VEGF and EGF families. The contribution of each pathway to carcinogenesis in obesity cannot be assessed in this nonmechanistic descriptive study, but the changes induced by surgery in this study seem sufficiently large to suggest that at least some of the molecular pathways are being deactivated, presumably by reducing the adipose tissue mass and other factors such as reduced insulin and insulin receptor signaling.

The current understanding of the relationship between excess body fat and cancer is that metabolically active visceral adipose tissue induces an upregulation of the molecular pathways involved in inflammation, insulin resistance, angiogenesis, and the expression of adipokines and other growth factors [11, 12]. As many of the downstream products of these pathways have been variously implicated in providing a tissue microenvironment favorable for the development of cancer, alterations following bariatric surgery offer insights into the reversibility of some of these processes. Consequently, it should be possible to demonstrate biological plausibility for the reduction of cancer risk and mortality observed in clinical studies [8–10].

In our panel of 34 cancer-associated plasma biomarkers, 12 have been implicated in angiogenesis. Silha et al. [25] have previously reported that, of the angiogenic biomarkers relevant to our study, VEGF-A, VEGF-C, VEGF-D, sVEGFR2, and angiopoietin-2 are elevated in obese subjects compared to normal controls. We showed a significant decrease in all of the VEGF family of growth factors studied, including PLGF, but not their corresponding receptors following laparoscopic sleeve gastrectomy (LSG). This expands on a previous study that demonstrated a reduction in VEGF-A up to 1 year following vertical banded gastroplasty, gastric bypass, and biliopancreatic diversion [17]. In addition, we observed a significant reduction in endoglin, a transmembrane accessory receptor for the cytokine TGF-β. Endoglin stimulates proliferation and migration of vascular endothelial cells in vivo via activation of the activin receptor-like kinase 1 (ALK1) pathway [26]. Increased expression has been demonstrated in the angiogenic endothelial cells of human tumor samples [27] and correlated with the high white adipose tissue burden in obese rodent models [28]. Considered together, these results suggest that the proangiogenic cascade is reversible and this change occurs early after bariatric surgery.

Of the ligands that bind to the epidermal growth factor receptor (EGFR) family of receptor tyrosine kinases associated with cell proliferation [29], we observed a decrease in EGF, TGFα, and heparin-binding EGF-like growth factor (HBEGF). Neither sEGFR (ErbB) nor sHER2/neu (ErbB2), their corresponding soluble receptors, were significantly altered. EGF, TGFα, and HBEGF have all been associated with cancer. Of these, HBEGF is abundantly expressed in human adipose tissue [30], TGFα has been implicated in the development of obesity-related postmenopausal breast cancer using rodent models [31], and EGF has received much attention as a therapeutic target in anticancer therapies [32]. If the production of these ligands is upregulated in obesity, there are significant implications for EGFR-mediated cell proliferation, invasion, and metastasis of epithelial malignancies via downstream activation of the ERK, PI3 kinase/AKT, and JAK/STAT pathways [32, 33]. Expression of TGFα, EGF, and EGFR worsens prognosis and increases the metastatic potential of breast [34], renal [35], and colorectal cancers [36], all known to be obesity-associated malignancies. Altered expression of these ligands has not been previously reported following bariatric surgery.

The chronic, low-grade inflammatory state associated with obesity is strongly suspected to play an integral role in the development of malignancy. The upregulation of inflammatory cytokines in obesity has been extensively investigated and is thought to be related to the production of adipokines [37], infiltration of inflammatory immune cells [38], and tissue hypoxia associated with the proliferation of adipose tissue [39]. The inflammatory mediators and adipokines IL-6, IL-8, and TNFα were significantly reduced in our investigation, confirming the findings of previous bariatric surgery studies, including LSG [14, 21, 40]. All three have been implicated in tumor proliferation, angiogenesis, and metastasis via activation of multiple upstream pathways, including STAT3 [41], CXCR1 and CXCR2 [42], and NF-κB [43], respectively. Additionally, the SERPINE1 gene transcript PAI-1, an inflammatory cytokine also implicated in angiogenesis and metastasis through extracellular matrix remodeling [44], was significantly decreased following bariatric surgery in our study. Decreased expression has similarly been demonstrated up to 6 months following LSG [20].

The inflammatory-related proteins should be considered together with the less well-established markers of inflammation included in our panel, such as FasL and sCD40L. The novel finding of significantly reduced Fas-ligand (FasL) levels reported here is linked to obesity; increased expression of FasL has been associated with adipocyte hypertrophy and macrophage infiltration in adipose tissue [45], for example. FasL is known to induce apoptosis by binding the death receptor Fas (CD95) [46] but may have an additional nonapoptotic inflammatory role via induction of gene expression for the ERK, JNK, and NF-κB pathways [47], in addition to increasing expression of the IL-1β, IL-6, and MCP-1 cytokines [48]. The cytokine sCD40L was also significantly reduced with surgical weight loss. In vivo studies of human cancers have demonstrated higher levels of the cytokine sCD40L secondary to tumor-induced platelet activation [49]. Traditionally, this was thought to promote angiogenesis via activation of the CD40/CD40L pathway [49], but more recently, an immunosuppressive effect secondary to inflammation has been proposed [50]. The observed reduction of sCD40L in our study would support both hypotheses given the reduction in the other proangiogenic biomarkers studied and the known secondary immunosuppression caused by inflammation in obesity [51].

It is plausible that the results of our study may be alternatively explained by a resolution of insulin resistance following LSG. Although most of the patients did not have insulin resistance or T2DM, it is well known that raised systemic insulin levels can potentiate anabolic effects to mediate cell proliferation and enhance tumor growth [52]. Related to this hypothesis, our finding of decreased IGFBP-1 following LSG is of uncertain significance. A recent review [53] found that there is a lack of evidence for IGFBP-1 stimulating tumor growth or cell migration, and that it may in fact play a role in tumor growth inhibition.

Limitations of the present study include the relatively small sample size. A study with later follow-up may also find more statistically significant changes. The nonsignificant result for leptin, for example, is at odds with similar bariatric surgery studies that included more patients [14]. The present study also lacks the large cohort of healthy weight (BMI < 25 kg/m^2^) controls that would be required, at a considerable reagent cost, to establish normal values for these biomarkers. Of the cancer-associated biomarkers we have measured, the National Cancer Institute has approved only four for routine clinical use in the context of breast cancer: mutations in EGFR, HER2/neu gene overexpression, as well as uPA and PAI-1 [54]. Areas for future study include the ongoing elucidation of the biological function of these gene products and pathways in healthy and overweight or obese individuals, longer term follow-up, and a comparison of the expression human cancer biomarkers in obese and healthy weight patients undergoing nonbariatric surgery.

## Conclusion

Effective weight loss, which is most reliably provided in obese individuals by bariatric surgery, results in a rapid decrease in the measured levels of cancer-associated circulating proteins. This suggests that obesity-associated carcinogenic processes may be directly attenuated by bariatric surgery, which can thus be considered to provide a cancer prevention role in the obese.

## Abbreviations

EGF: Epidermal growth factor
FGF-basic: Basic fibroblast growth factor
G-CSF: Granulocyte colony-stimulating factor
HB-EGF: Heparin-binding EGF-like growth factor
HGF: Hepatocyte growth factor
IGFBP-1: Insulin-like growth factor binding protein-1
IL-6: Interleukin-6
IL-8: Interleukin-8
IL-18: Interleukin-18
LSG: Laparoscopic sleeve gastrectomy
PAI-1: Plasminogen activator inhibitor-1
PDGF-AB/AA: Platelet-derived growth factor-AB/AA
PECAM-1: Platelet endothelial cell adhesion molecule-1
PLGF: Placental growth factor
sCD40L: Soluble CD40 ligand
SCF: Stem cell factor
sEGFR: Soluble epidermal growth factor receptor
sFASL: Soluble Fas ligand receptor
sHER-2/neu: Soluble human epidermal growth factor 2
sIL-6Rα: Soluble interleukin-6 receptor alpha
sTIE-2: Soluble Tie2 receptor
sVEGFR-1: Soluble vascular endothelial growth factor receptor 1
sVEGFR-2: Soluble vascular endothelial growth factor receptor 2
TGFα: Transforming growth factor alpha
TNFα: Tumor necrosis factor alpha
uPA: Urokinase plasminogen activator
VEGF-A: Vascular endothelial growth factor-A
VEGF-C: Vascular endothelial growth factor-C
VEGF-D: Vascular endothelial growth factor-D

## References

[1] Olshansky SJ, Passaro DJ, Hershow RC (2005). A potential decline in life expectancy in the United States in the twenty-first century. N Engl J Med.

[2] World Health Organization. Global status report on noncommunicable diseases 2014. Geneva, 2015. 10.1161/STROKEAHA.115.008097 25873596

[3] Calle EE, Rodriguez C, Walker-Thurmond K (2003). Overweight, obesity, and mortality from cancer in a prospectively studied cohort of U.S. adults. N Engl J Med.

[4] Renehan AG, Tyson M, Egger M (2008). Body-mass index and incidence of cancer: a systematic review and meta-analysis of prospective observational studies. Lancet.

[5] Jemal A, P, Bray F, Torre L, Forman L, eds. The Cancer Atlas. 2nd ed. Atlanta, GA: American Cancer Society; 2015.

[6] Buchwald H, Avidor Y, Braunwald E (2004). Bariatric surgery: a systematic review and meta-analysis. JAMA.

[7] Colquitt JL, Pickett K, Loveman E (2014). Surgery for weight loss in adults. Cochrane Database Syst Rev.

[8] Adams TD, Gress RE, Smith SC (2007). Long-term mortality after gastric bypass surgery. N Engl J Med.

[9] Christou NV, Lieberman M, Sampalis F (2008). Bariatric surgery reduces cancer risk in morbidly obese patients. Surg Obes Relat Dis.

[10] Sjostrom L, Gummesson A, Sjostrom CD (2009). Effects of bariatric surgery on cancer incidence in obese patients in Sweden (Swedish Obese Subjects Study): a prospective, controlled intervention trial. Lancet Oncol.

[11] Khandekar MJ, Cohen P, Spiegelman BM (2011). Molecular mechanisms of cancer development in obesity. Nat Rev Cancer.

[12] Park J, Morley TS, Kim M (2014). Obesity and cancer-mechanisms underlying tumour progression and recurrence. Nat Rev Endocrinol.

[13] Pories WJ, Swanson MS, MacDonald KG (1995). Who would have thought it? An operation proves to be the most effective therapy for adult-onset diabetes mellitus. Ann Surg.

[14] Samaras K, Viardot A, Botelho NK (2013). Immune cell-mediated inflammation and the early improvements in glucose metabolism after gastric banding surgery. Diabetologia.

[15] Schauer PR, Bhatt DL, Kirwan JP (2014). Bariatric surgery versus intensive medical therapy for diabetes--3-year outcomes. N Engl J Med.

[16] Vázquez LA, Pazos F, Berrazueta JR (2005). Effects of changes in body weight and insulin resistance on inflammation and endothelial function in morbid obesity after bariatric surgery. J Clin Endocrinol & Metab..

[17] Garcia de la Torre N, Rubio MA, Bordiu E (2008). Effects of weight loss after bariatric surgery for morbid obesity on vascular endothelial growth factor-A, adipocytokines, and insulin. J Clin Endocrinol & Metab..

[18] Klein S, Mittendorfer B, Eagon JC (2006). Gastric bypass surgery improves metabolic and hepatic abnormalities associated with nonalcoholic fatty liver disease. Gastroenterology.

[19] Schernthaner GH, Kopp HP, Kriwanek S (2006). Effect of massive weight loss induced by bariatric surgery on serum levels of interleukin-18 and monocyte-chemoattractant-protein-1 in morbid obesity. Obes Surg.

[20] Umemura A, Sasaki A, Nitta H (2014). Effects of changes in adipocyte hormones and visceral adipose tissue and the reduction of obesity-related comorbidities after laparoscopic sleeve gastrectomy in Japanese patients with severe obesity. Endocr J.

[21] Viana EC, Araujo-Dasilio KL, Miguel GP (2013). Gastric bypass and sleeve gastrectomy: the same impact on IL-6 and TNF-alpha. Prospective clinical trial. Obes Surg.

[22] Khan A (2012). Detection and quantitation of forty eight cytokines, chemokines, growth factors and nine acute phase proteins in healthy human plasma, saliva and urine. J Proteome.

[23] Schernthaner GH, Kopp HP, Krzyzanowska K (2006). Soluble CD40L in patients with morbid obesity: significant reduction after bariatric surgery. Eur J Clin Investig.

[24] Mingrone G, Manco M, Iaconelli A (2008). Prolactin and insulin ultradian secretion and adipose tissue lipoprotein lipase expression in severely obese women after bariatric surgery. Obesity (Silver Spring).

[25] Silha JV, Krsek M, Sucharda P (2005). Angiogenic factors are elevated in overweight and obese individuals. Int J Obes (2005).

[26] Lebrin F, Goumans MJ, Jonker L (2004). Endoglin promotes endothelial cell proliferation and TGF-beta/ALK1 signal transduction. EMBO J.

[27] Miller DW, Graulich W, Karges B (1999). Elevated expression of endoglin, a component of the TGF-beta-receptor complex, correlates with proliferation of tumor endothelial cells. Int J Cancer.

[28] Kurki E, Shi J, Martonen E (2012). Distinct effects of calorie restriction on adipose tissue cytokine and angiogenesis profiles in obese and lean mice. Nutr Metabolism.

[29] Cho HS, Mason K, Ramyar KX (2003). Structure of the extracellular region of HER2 alone and in complex with the Herceptin Fab. Nature.

[30] Matsumoto S, Kishida K, Shimomura I (2002). Increased plasma HB-EGF associated with obesity and coronary artery disease. Biochem Biophys Res Commun.

[31] Cleary MP, Phillips FC, Getzin SC (2003). Genetically obese MMTV-TGF-alpha/Lep(Ob)Lep(Ob) female mice do not develop mammary tumors. Breast Cancer Res Treat.

[32] Henson ES, Gibson SB (2006). Surviving cell death through epidermal growth factor (EGF) signal transduction pathways: implications for cancer therapy. Cell Signal.

[33] Normanno N, De Luca A, Bianco C (2006). Epidermal growth factor receptor (EGFR) signaling in cancer. Gene.

[34] Umekita Y, Ohi Y, Sagara Y (2000). Co-expression of epidermal growth factor receptor and transforming growth factor-alpha predicts worse prognosis in breast-cancer patients. Int J Cancer.

[35] Uhlman DL, Nguyen P, Manivel JC (1995). Epidermal growth factor receptor and transforming growth factor alpha expression in papillary and nonpapillary renal cell carcinoma: correlation with metastatic behavior and prognosis. Clin Cancer Res.

[36] Yokoi K, Thaker PH, Yazici S (2005). Dual inhibition of epidermal growth factor receptor and vascular endothelial growth factor receptor phosphorylation by AEE788 reduces growth and metastasis of human colon carcinoma in an orthotopic nude mouse model. Cancer Res.

[37] Lysaght J, van der Stok EP, Allott EH (2011). Pro-inflammatory and tumour proliferative properties of excess visceral adipose tissue. Cancer Lett.

[38] Nishimura S, Manabe I, Nagasaki M (2009). CD8+ effector T cells contribute to macrophage recruitment and adipose tissue inflammation in obesity. Nat Med.

[39] Halberg N, Khan T, Trujillo ME (2009). Hypoxia-inducible factor 1alpha induces fibrosis and insulin resistance in white adipose tissue. Mol Cell Biol.

[40] Illan-Gomez F, Gonzalvez-Ortega M, Orea-Soler I (2012). Obesity and inflammation: change in adiponectin, C-reactive protein, tumour necrosis factor-alpha and interleukin-6 after bariatric surgery. Obes Surg.

[41] Bromberg JF, Wrzeszczynska MH, Devgan G (1999). STAT3 as an oncogene. Cell.

[42] Schraufstatter IU, Chung J, Burger M (2001). IL-8 activates endothelial cell CXCR1 and CXCR2 through rho and Rac signaling pathways. Am J Phys.

[43] Pikarsky E, Porat RM, Stein I (2004). NF-kappaB functions as a tumour promoter in inflammation-associated cancer. Nature.

[44] Dano K, Behrendt N, Hoyer-Hansen G (2005). Plasminogen activation and cancer. Thromb Haemost.

[45] Bluher M, Kloting N, Wueest S (2014). Fas and FasL expression in human adipose tissue is related to obesity, insulin resistance, and type 2 diabetes. J Clin Endocrinol & Metab.

[46] Waring P, Mullbacher A (1999). Cell death induced by the Fas/Fas ligand pathway and its role in pathology. Immunol Cell Biol.

[47] Matsumoto N, Imamura R, Suda T (2007). Caspase-8- and JNK-dependent AP-1 activation is required for Fas ligand-induced IL-8 production. FEBS J.

[48] Wajant H, Pfizenmaier K, Scheurich P (2003). Non-apoptotic Fas signaling. Cytokine Growth Factor Rev.

[49] Roselli M, Mineo TC, Basili S (2004). Soluble CD40 ligand plasma levels in lung cancer. Clin Cancer Res.

[50] Huang J, Jochems C, Talaie T (2012). Elevated serum soluble CD40 ligand in cancer patients may play an immunosuppressive role. Blood.

[51] Okwan-Duodu D, Umpierrez GE, Brawley OW (2013). Obesity-driven inflammation and cancer risk: role of myeloid derived suppressor cells and alternately activated macrophages. Am J Cancer Res.

[52] Johnson JA, Gale EA (2010). Diabetes, insulin use, and cancer risk: are observational studies part of the solution-or part of the problem?. Diabetes.

[53] Baxter RC (2014). IGF binding proteins in cancer: mechanistic and clinical insights. Nat Rev Cancer.

[54] National Cancer Institute. Tumor Markers Fact Sheet. Retrieved 13 Apr 2016 from: http://www.cancer.gov/about-cancer/diagnosis-staging/diagnosis/tumor-markers-fact-sheet.

